# Mesenchymal Stromal Cell-Derived Extracellular Vesicles as Biological Carriers for Drug Delivery in Cancer Therapy

**DOI:** 10.3389/fbioe.2022.882545

**Published:** 2022-04-14

**Authors:** María Cecilia Sanmartin, Francisco Raúl Borzone, María Belén Giorello, Gustavo Yannarelli, Norma Alejandra Chasseing

**Affiliations:** ^1^ Laboratorio de Inmunohematología, Instituto de Biología y Medicina Experimental (IBYME), Consejo Nacional de Investigaciones Científicas y Técnicas (CONICET), Buenos Aires, Argentina; ^2^ Laboratorio de Regulación Génica y Células Madre, Instituto de Medicina Traslacional, Trasplante y Bioingeniería (IMeTTyB), Universidad Favaloro - CONICET, Buenos Aires, Argentina

**Keywords:** mesenchymal stem/ stromal cells, extracellular vesicles, cancer therapy, drug delivery systems, cell-free therapy

## Abstract

*Cancer* is the second leading cause of death worldwide, with 10.0 million cancer deaths in 2020. Despite advances in targeted therapies, some pharmacological drawbacks associated with anticancer chemo and immunotherapeutic agents include high toxicities, low bioavailability, and drug resistance. In recent years, extracellular vesicles emerged as a new promising platform for drug delivery, with the advantage of their inherent biocompatibility and specific targeting compared to artificial nanocarriers, such as liposomes. Particularly, mesenchymal stem/stromal cells were proposed as a source of extracellular vesicles for cancer therapy because of their intrinsic properties: high *in vitro* self-renewal and proliferation, regenerative and immunomodulatory capacities, and secretion of extracellular vesicles that mediate most of their paracrine functions. Moreover, extracellular vesicles are static and safer in comparison with mesenchymal stem/stromal cells, which can undergo genetic/epigenetic or phenotypic changes after their administration to patients. In this review, we summarize currently reported information regarding mesenchymal stem/stromal cell-derived extracellular vesicles, their proper isolation and purification techniques - from either naive or engineered mesenchymal stem/stromal cells - for their application in cancer therapy, as well as available downstream modification methods to improve their therapeutic properties. Additionally, we discuss the challenges associated with extracellular vesicles for cancer therapy, and we review some preclinical and clinical data available in the literature.

## Introduction

*Cancer* is the second leading cause of death worldwide after cardiovascular diseases (Cortes et al., 2020; Lih et al., 2020). The GLOBOCAN cancer statistics estimated 19.3 million new cases and 10 million deaths worldwide in 2020 (Ferlay et al., 2021). Despite advances in targeted therapies and immunotherapies, tumors may not only develop drug resistance–in response to therapy or due to intrinsic intratumoral heterogeneity - and metastasize to distant organs, but also many patients do not benefit from the currently available therapies, or they suffer from off-target or immune-related adverse effects (Martin et al., 2020; Saber et al., 2020). Moreover, some pharmacological difficulties are associated with anticancer chemotherapeutic agents, such as elevated toxicities, low bioavailability of free drugs, and drug resistance (Fang et al., 2020; Parodi et al., 2021). In consequence, nanodrug delivery systems–mainly liposomes - have been studied as an attractive option due to their several advantages, which include controlled drug release, protection from degradation in the circulation, reduced side effects, and increased drug solubility (Fang et al., 2020; van der Koog et al., 2021). However, artificial nanocarriers exert some limitations, such as rapid plasma clearance and accumulation in clearance organs, immunogenicity, unspecific targeting, and hypersensitivity reactions (De Jong et al., 2019; Attia and Mashal, 2021; van der Koog et al., 2021). In recent years, extracellular vesicles (EVs) have gained recognition as a new promising platform for drug delivery, with the advantage of their inherent biocompatibility because of their complex biological composition compared to artificial nanocarriers (van der Koog et al., 2021). Remarkably, mesenchymal stem/stromal cells (MSCs) emerged as a source of EVs for cancer therapy because of their inherent characteristics, with EVs either obtained from naïve or genetically engineered MSCs. In the next sections, we will discuss the properties that make MSCs suitable parental cells for the isolation of therapeutic EVs, as well as EVs manufacturing process and methods employed for their modification, with their respective benefits and limitations. Finally, we will review some pre-clinical and clinical data published in the last 5 years regarding EVs as drug delivery systems (DDSs) for cancer therapy.

## General Characteristics of Mesenchymal Stem/Stromal Cells and Their Relevance in Cell Therapy

MSCs are non-hematopoietic multipotent precursor cells, first discovered by Friedenstein and colleagues in the bone marrow (BM) stroma, that contribute to the maintenance and regeneration of different connective tissues (such as bone, cartilage, adipose, and muscle tissues) (Gregory et al., 2005; Nombela-Arrieta et al., 2011). These cells can differentiate into multiple mesoderm-type cell lineages, like osteoblasts, chondrocytes, and endothelial cells, and non-mesoderm-type lineages, such as neuronal-like cells (Kassem et al., 2004). In addition, MSCs exhibit a high clonogenic nature, with rapid *in vitro* expansion and development of fibroblasts colony-forming units (Prockop and Oh, 2012), desirable properties when culturing parental cells for EVs isolation. In accordance with the criteria defined by the Mesenchymal and Tissue Stem Cell Committee of the International Society for Cellular Therapy, human MSCs are distinguished based on some minimal characteristics. These include their plastic-adherence, the expression of 95–99% levels of CD73, CD90, and CD105 surface markers, as well as the lack of expression of human leukocyte antigen (HLA)-DR, CD34, CD45, CD14 or CD11b, CD79a or CD19 markers, and also the ability to differentiate into adipogenic, chondrogenic and osteogenic lineages *in vitro* (Dominici et al., 2006; Brown et al., 2019). Because of their low expression of major histocompatibility complex (MHC) type I and lack of expression of MHC II molecules, MSCs are considered an immunoprivileged cell type, with a low risk of cellular rejection when used for cellular therapy (Lee and Hong, 2017). Apart from BM, MSCs can be isolated from other tissues, including adipose tissue (AT), dental pulp, umbilical cord (UC) blood and perivascular tissue, as well as from menstrual blood (Berebichez-Fridman and Montero-Olvera, 2018). While BM-MSCs are more difficult to obtain since they involve performing painful procedures to patients, AT-MSCs are relatively easy to isolate from AT available as a subproduct of cosmetic procedures (i.e., liposuction), while exhibiting similar phenotypic and functional properties to BM-MSCs (Berebichez-Fridman and Montero-Olvera, 2018). Regarding the self-renewal potential and proliferation capacity, MSCs may differ in these properties depending on their source of origin (Brown et al., 2019). Both human UC perivascular cells (HUCPVCs) and AT-MSCs have higher proliferation rates than BM-MSCs, being an attractive option to the latter for cell therapy (Kern et al., 2006; Yannarelli et al., 2013). Particularly, HUCPVCs overcome BM-MSCs limitation of reduced proliferation potential with increasing donor age and the increased heterogeneity between donors (Yannarelli et al., 2013).

Regarding their functional properties, MSCs are well known for their immunomodulatory effects both *in vitro* and *in vivo*. These cells secrete a variety of soluble factors and chemokines that mediate immunosuppression by inhibiting B and T cells, monocyte maturation, as well as the generation of regulatory T cells and M2 macrophages polarization (Nauta and Fibbe, 2007; Kim et al., 2013; de Witte et al., 2015; Jiang and Xu, 2020). Moreover, depending on the signals of the particular microenvironment, BM-MSCs can polarize into two different subtypes upon the stimulation of Toll-like receptors 3 or 4 (He et al., 2009). MSC1 or toll-like receptor 4-stimulated MSCs exhibit a pro-inflammatory phenotype, as they secrete the C-X-C Motif Chemokine Ligand (CXCL) 1, interleukin (IL)-6, IL-8 -among others-, whereas MSC2—or toll-like receptor 3-stimulated MSCs - secrete immunosuppressive factors, such as prostaglandin E2, indoleamine-2,3-dioxygenase, and IL-10 (Meisel et al., 2004; Tomchuck et al., 2008; de Witte et al., 2015). In addition to their immunomodulatory properties, BM-MSCs can mobilize, through peripheral circulation, to injured tissues during the healing process (Fu et al., 2019). Among the factors that regulate the migration of BM-MSCs, there are chemical factors, including CXCL12 chemokine, osteopontin, vascular endothelial growth factor, insulin growth factor 1, and transforming growth factor-beta, as well as mechanical factors such as mechanical strain, shear stress, matrix stiffness and microgravity (Fu et al., 2019). Once BM-MSCs reach the injury site, they can establish cell-to-cell contacts or secrete many bioactive factors that promote suppression of local immune responses, stimulation of fibrotic tissue formation, and modulation of angiogenesis and cell proliferation (Samsonraj et al., 2017).

During the last 20 years, MSCs have emerged as a promising source for cell therapy to treat several disorders because of their immunomodulatory and regenerative properties. While the first clinical trials with MSCs included osteogenesis imperfecta and graft vs. host disease (Horwitz et al., 1999; Lee et al., 2009), soon after, these cells began to be studied to treat immune-mediated and degenerative disorders (Saeedi et al., 2019). These pathologies include ischemia-reperfusion induced injury (Zhang et al., 2020), myocardial infarction (Ward et al., 2018), covid-19 pneumonia (Metcalfe, 2020), and cancer (Hmadcha et al., 2020). As Saeedi et al. reviewed in 2019, autoimmune diseases (25%), cardiovascular diseases (15%), and neuro degenerative diseases (12%) were the top-three diseases studied in clinical trials involving the administration of MSCs (Saeedi et al., 2019). Regarding MSCs use for treating cardiovascular diseases, these cells are known to have protective effects on the myocardium, by reducing inflammation and promoting angiogenesis and apoptosis resistance (Guo et al., 2020). However, their systemic administration caused embolism and inflammation, according to the clinical trials reports (Guo et al., 2020). Additionally, MSCs properties such as their homing capacity, immunomodulation, inhibition of inflammation and their ability to differentiate into neuron-like cells under specific *in vitro* conditions, have been studied for the treatment of neurodegenerative diseases (Yao et al., 2020). Recently, MSCs have been also tested in several clinical trials to treat the adverse effects caused by the SARS-CoV-2 infection, via their immunomodulatory and anti-inflammatory effects (Moradinasab et al., 2021).

With reference to the most suitable source of MSCs for cell-therapy, BM-MSCs are known to exhibit tropism for tumor sites and decreased immunosuppression, whereas AT-MSCs and UC-MSCs exert higher proliferation rates and are easier to isolate (Li et al., 2014). Additionally, both human UC-MSCs and AT-MSCs showed tropism toward cancer cells *in vitro* (Gondi et al., 2010; Chen et al., 2019). As Pulukuri et al. reported, the induction of urokinase plasminogen activator expression by histone deacetylase inhibitors could represent a strategy for enhancing the tumor tropism of MSCs (Pulukuri et al., 2010). Although nowadays it is still unclear which source of MSCs is better for therapeutic applications, we hypothesize that UC-MSCs could represent the best option for their use in cell-therapy, particularly in cancer, due to the mentioned properties.

Apart from using naïve MSCs for therapeutic applications, these cells can be modified by pre-conditioning with specific factors or employing genetic engineering techniques to enhance their therapeutic properties and/or reduce potential disadvantages (i.e., improvement of adhesion and survival or preventing premature senescence) (Wiredu Ocansey et al., 2020). For instance, human adult MSCs can be modified to deliver pro-apoptotic proteins or cytokines, such as tumor necrosis factor related apoptosis induced ligand (TRAIL), with positive anti-tumoral effects on colorectal cancer, or IL-12 overexpressing murine BM-MSCs that induced tumor cells apoptosis in melanoma and lung cancer murine models (Chulpanova et al., 2018). Additionally, an ongoing clinical trial (NCT03298763) is testing the administration of TRAIL-modified MSCs in combination with cisplatin/pemetrexed chemotherapy in metastatic non-small cell lung cancer patients. Moreover, it is easier to genetically modify MSCs than hematopoietic stem cells, while MSCs retain their *in vivo* activity after their modification (Attia and Mashal, 2021). Developing new and improved transfection methods –both chemical and physical - without associated risks –such as insertional mutations or adverse immune reactions –may facilitate those procedures (Attia and Mashal, 2021).

Despite those beneficial properties, the role of MSCs in cancer progression remains contradictory. On the one hand, a considerable amount of evidence supports the fact that BM-MSCs can migrate to primary tumors where the microenvironment educates them to become pro-tumoral, either as tumor-associated MSCs, or differentiated into tumor-associated fibroblasts as well (Hill et al., 2017; Giorello et al., 2021). In this way, BM-MSCs can promote tumor growth, immunosuppression, inflammation, drug resistance, angiogenesis, invasion and metastasis, and may even promote the establishment of pre-metastatic niches in distant organs (Gao et al., 2018; Pietrovito et al., 2018; Xu et al., 2018; Sanmartin et al., 2021). On the other hand, some studies showed that MSCs could exert inhibitory effects on tumor cells. For example, it was described that murine BM-MSCs inhibited tumor cell growth *in vitro* and *in vivo* in a cell number-dependent manner by inducing cell cycle arrest and apoptotic death in different cancer cell lines (Lu et al., 2008). Similarly, Bruno S. et al. also showed that human BM-MSC-derived EVs induced cell cycle arrest and inhibited tumor growth of hepatoma, Kaposi´s sarcoma, and ovarian cancer cell lines *in vivo* (Bruno et al., 2014). Li et al. published some contradictory results, showing that human BM-MSCs promoted the proliferation of hepatocellular carcinoma cells *in vitro*, and significantly inhibited invasiveness and metastasis through downregulation of transforming growth factor-beta (Li et al., 2010). Likewise, Bajetto et al. demonstrated *in vitro* that UC-MSCs anti-tumoral effects on glioblastoma cells were mediated by direct cell-to-cell contacts, while pro-tumoral effects involved releasing soluble factors (Bajetto et al., 2017). Moreover, Zhu et al. identified factor Dickkopf-1 as a key molecule - secreted byAT-MSCs - that may mediate the inhibitory effect of leukemia cells proliferation through the negative regulation of the WNT signaling pathway (Zhu et al., 2009). The evident discrepancies between studies may be due to the heterogeneity in MSCs sources and donors, as well as differences between types of cancer, cancer cell lines and *in vivo* models, and the lack of standardized culturing methods.

Apart from this controversy around the effects of MSCs on cancer cells, culture-expanded MSCs could potentially undergo malignant transformation and senescence-associated modifications at specific CpG sites (Wang et al., 2012a; 2012b). Senescent MSCs exert an altered phenotype, with decreased immunological properties and a higher production of pro-inflammatory cytokines, such as IL-6 and IL-8 (Drela et al., 2019). Nevertheless, it was subsequently demonstrated that MSCs exerting these abnormalities neither exhibited a growth advantage *in vitro* nor led to tumor formation in immunocompromised mice (Rühle et al., 2019). According to the exclusion criteria proposed by the Cell Products Working Party and the Committee for Advanced Therapies, MSCs must be discarded upon identifying two identical abnormal metaphases on 20 metaphases in a karyotyping analysis (Rühle et al., 2019). However, even though no patients enrolled in clinical trials of MSC-therapies have been diagnosed with cancer (Lee and Hong, 2017), further studies need to be accomplished to assess this potential adverse effect in-depth. Furthermore, Di et al. demonstrated that *in vitro* aged human UC-MSCs (at passage 45) significantly promoted *in vitro* proliferation and migration of breast cancer cells and *in vivo* tumor progression compared with their ‘young’ counterparts (UC-MSCs at passage 5). They also identified IL-6 as the main mediator of these pro-tumoral effects (Di et al., 2014). It has also been shown that neonatal MSCs have higher proliferation potential in comparison to MSCs derived from adult sources (Drela et al., 2019). This evidence suggests that aged MSCs –or MSCs from aged donors - could represent a risk when used as anti-cancer cell therapies.

Another mechanism associated with MSC-based therapies is mitochondrial transfer. BM-MSCs are able to donate mitochondria to rescue cells from tissue damage through tunneling nanotubes (TNT), gap-junctions, or mitochondrial DNA transfer through EVs (Li et al., 2019a). In addition, MSCs from different sources may alter oxidative phosphorylation and reactive oxygen species generation through mitochondrial transfer, which leads to acute and chronic inflammation and apoptotic cell death (Stavely and Nurgali, 2020). However, mitochondrial transfer-based therapies could be disadvantageous in cancer patients since BM-MSCs that migrate to the primary tumor could transfer mitochondria to cancer cells, enhancing their chemo-resistance and proliferation rates (Pasquier et al., 2013). In this way, some investigators showed that the inhibition of TNT contacts –through Intercellular Adhesion Molecule 1 blocking - between UC-MSCs and human acute T cell leukemia Jurkat cells, lead to chemotherapy-induced tumor cell death (Paliwal et al., 2018), positioning TNT inhibition as a novel target therapy in some types of cancer. Finally, the therapeutic effect of MSCs in chemotherapy-induced tissue damage has also been studied *in vitro*. MSCs –independently of their tissue of origin- are radioresistant cells, as well as they appear to be unaffected by some chemotherapeutic agents commonly used for cancer treatment. This may be due to their high expression of anti-apoptotic proteins and their elevated antioxidant activity (Rühle et al., 2019). Further *in vitro* and *in vivo* studies are required to elucidate the mechanisms by which MSCs exert regeneration in the context of chemotherapy-induced injuries before the clinical translation of those results.

## Extracellular Vesicles as a New Paradigm for Cell-Free Therapy

Despite the fact that MSCs have been used in many clinical trials to treat several pathologies, the inoculation of viable MSCs into patients entails some hindrances. These include limited passaging before entering a senescent state –in particular with BM-MSCs -, cellular heterogeneity, the alteration of their differentiation potential under hypoxic conditions, the occurrence of epigenetic changes during culturing - in genes that regulate self-renewal -, excessive immunosuppression, and risk of lung embolism (Lee et al., 2009; Brown et al., 2019; Wang et al., 2020). Those are the main reasons why MSC-derived EVs have arisen as an interesting and potential option to MSCs for the treatment of several diseases, including cartilage defects, osteoarthritis, renal injury, hepatocellular injury, macular degeneration, and diabetes mellitus (Cai et al., 2020; Rostom et al., 2020). Since the discovery of EVs therapeutic properties, the number of published articles reporting their potential applications for the treatment of different pathologies raised from a few tens in 2015 to over 1,000 in 2021 (Racchetti and Meldolesi, 2021), thus reinforcing EVs potentiality when used as cell-free therapy. In addition, many groups previously described that MSCs mediate their biological and regenerative effects through the secretion of EVs (Phelps et al., 2018; Witwer et al., 2019), and that MSC-derived EVs seem to have the same effects as the parental cells while considered safer (Gratpain et al., 2021). Moreover, it was reported that MSC-derived EVs isolated from MSCs of different sources might be beneficial for treating specific diseases. For instance, BM-MSC-derived EVs exerted good properties for treating cartilage defects or osteoarthritis, due to their intrinsic bone tropism, while AT-MSC-derived EVs regulate inflammation in various disease models (Cai et al., 2020). Human UC-MSC-derived EVs were shown to promote neural restoration, heart repair, and protection of liver and kidney, through promoting angiogenesis and reducing apoptosis, as well as these cells are known to produce higher amounts of EVs when compared with MSCs from other sources (Cai et al., 2020). Particularly, using MSCs as parental cells for EVs isolation is advantageous since clinical-grade MSCs have been cultured for years for cell therapy applications, already meeting the regulatory requirements and following Good Manufacturing Practice (GMP) guidelines (Burnouf et al., 2019).

According to the International Society of Extracellular Vesicles, EVs are “nanosized lipid bilayer encapsulated membranes carrying proteins, lipids, nucleic acids and sugars that are shed by the majority of the cells into the extracellular milieu to mediate intercellular communication, by transferring molecules from parental/donor cells to target cells” (Soekmadji et al., 2020). Additionally, EVs can be classified based on their size, origin, and content (Battistelli and Falcieri, 2020). Exosomes are 30–200 nm vesicles originated from the endosomal compartment, secreted when multivesicular endosomes fuse with the cell membrane releasing their intraluminal vesicles in the extracellular space (Van Niel et al., 2018). Exosomes also have a complex membrane composition, consisting of a lipid bilayer with cholesterol and sphingomyelin present in lipid rafts. They can transport different molecular cargos –such as proteins, lipids, messenger RNAs (mRNAs) and microRNAs (miRNAs) - to target cells in both physiological and pathological contexts (Logozzi et al., 2019). Because of their endosomal origin, exosomes contain membrane transport and fusion proteins, tetraspanins (CD9, CD63, CD81), and proteins involved in the multivesicular bodies biogenesis: programmed cell death 6 interacting protein (Alix) and Tumor susceptibility gene 101 (TSG101). These proteins are widely used as exosome positive markers, although their presence may vary depending on exosomes origin (Vlassov et al., 2012). Alternatively, microvesicles (MVs) are nanosized particles - 50–1,000 nm in diameter - generated through cell membrane budding after agonist activation, shear/physical stress, or oxidative stress, and may contain organelles proteins –such as those from the endoplasmic reticulum, Golgi, mitochondria and nucleus—(Boilard, 2018; Agrahari et al., 2019; Bodega et al., 2019). MVs also participate in intercellular communication, modulating several biological functions (Zi-Tong et al., 2022). It is important to highlight that since analytical techniques do not differentiate between exosomes and MVs due to their range overlapping, the International Society for Extracellular Vesicles recommends using the term small EVs (sEVs) instead (Witwer and Théry, 2019).

Finally, apoptotic bodies (ABs) are the largest membrane-bound extracellular vesicles –of 1–5 μm in diameter - generated during apoptotic cells disassemble (Jiang et al., 2017). ABs play a relevant role in the clearance of apoptotic cells by phagocytes and in cell-to-cell communication through their molecular cargos (Jiang et al., 2017). These large EVs may contain chromatin, glycosylated proteins, RNA, and even entire organelles - mainly mitochondria and nuclear fragments - (Irimie and Berindan-neagoe, 2020). While previously neglected, more attention has been recently focused on ABs. For instance, it was reported that ABs might be involved in cancer progression and metastasis (Gregory and Dransfield, 2018; Irimie and Berindan-neagoe, 2020). Liu et al. demonstrated - in a myocardial infarction model - that transplanted murine BM-MSCs that undergo apoptosis can promote angiogenesis through the release of ABs, by regulating autophagy in recipient endothelial cells (Liu et al., 2020). Furthermore, Li et al. used ABs purified from AT-MSCs to improve skin wound healing (Li J. et al., 2022). It was recently elucidated that ABs represent a heterogeneous population, including ABs with a size of less than 1 µm –defined as apoptotic MVs - which differ in their physiological and membrane integrity properties from larger ABs (Kakarla et al., 2020).

Regarding EVs therapeutic benefits for cancer therapy, they have intrinsic and target-specific homing capabilities when compared to free drugs or artificial nanocarriers, which may be related to the presence of specific surface proteins - such as integrins - and their ability to penetrate biological barriers –like the blood-brain barrier - (Berumen Sánchez et al., 2021). EVs also protect biological cargo –especially miRNAs and mRNAs–from degradation after their administration *in vivo* (Famta et al., 2022). Moreover, modern techniques allow EVs modification to increase tissue targeting efficiency (Antes et al., 2018), as we will discuss later. Particularly, MSC-derived EVs may exert similar tumor-homing properties to their parental cells, as exosomes from UC-MSCs were shown to concentrate mainly in osteosarcoma tumors *in vivo* (Quadri et al., 2022). In addition, it was demonstrated that EVs could passively reach the tumor microenvironment through enhanced permeability and retention effect (Li H. et al., 2022). MSC-derived EVs also have lower immunogenicity than MSCs (Li et al., 2021). However, as evidence suggests, MSCs from different tissue sources may release EVs that differ in their functional properties (Cai et al., 2020). This has to be considered when choosing the source of parental MSCs for EVs isolation, depending on the specific therapeutic goal. Moreover, recent innovations applied to the engineering of EVs have progressively circumvented the limitations of naive EVs (Ullah et al., 2021), as we will discuss later.

However, nothing is ever as simple as it seems. Despite several EVs-associated advantages, many groups reported some disadvantages. For instance, because of their similarities in size, EVs may be contaminated with viruses, many of which can incorporate their genetic material or proteins into EVs –i.e., Epstein-Barr virus and Kaposi’s Sarcoma-Associated Herpesvirus - (McNamara and Dittmer, 2020). Moreover, the utilization of fetal bovine serum (FBS) for MSCs *in vitro* culturing not only represents a source of exogenous contaminating EVs, but also it was reported that FBS might alter MSCs phenotype, turning these cells immunogenic (Naskou et al., 2018; Lehrich et al., 2021). Additionally, it was shown that human BM-MSC-derived EVs might exert pleiotropic effects when administrated *in vivo* (Boulestreau et al., 2021). Finally, the cargo of BM-MSC-derived EVs can be highly dependent on cell culture conditions (Parfejevs et al., 2020). Heterogeneity in MSCs phenotype and secretome –including EVs - is also attributed to donor variability, variations in O_2_ tension, genetic manipulation, senescence and oxidative stress (Ratushnyy et al., 2020; Costa et al., 2021). This evidence supports the requirement of additional efforts for *in vitro* clinical-grade MSCs culturing standardization, following current GMP guidelines.

### Extracellular Vesicles Biodistribution and Tracking Assays

With reference to EVs biodistribution and pharmacokinetics upon their administration in animal models, evidence shows that they concentrate mainly in the liver, lungs, kidneys, and spleen (Kang et al., 2021). While most sEVs are located in the liver, larger EVs are concentrated mainly in the lungs (Kang et al., 2021). Differences between studies are attributed to the heterogeneity between EV sources, doses and routes of administration, animal models, chosen endpoints, as well as to mechanical factors –i.e., the more rigid the EVs are, the more efficient the uptake process is -, among others (Dang et al., 2020; Kang et al., 2021). Moreover, EVs clearance was reported to be rapid –between 1 and 6 h after administration - through renal and hepatic processing (Yin et al., 2020). This represents a limitation when using EVs as DDSs, since it may require the administration of multiple doses to achieve the desired therapeutic effect. Novel techniques are being developed in an attempt to improve EVs biodistribution and facilitate *in vivo* tracking. For example, Gangadaran et al. developed an *in vivo* bioluminescence imaging and tracking system for EVs based on *Renilla* luciferase (Gangadaran and Ahn, 2020). Similarly, Shimomura et al. designed novel fluorescent probes that bind EVs membranes - with no risk of EVs aggregation - for monitoring their uptake (Shimomura et al., 2021). Additional methods for non-invasive *in vivo* tracking of EVs include near-infrared dyes –with the advantages of their intense signal, low autofluorescence of biological tissue in the spectral range used, and deep tissue penetration of near-infrared light-, as well as EVs modified with ultra-small superparamagnetic iron oxide for their analysis by magnetic resonance imaging (Gangadaran et al., 2018). In addition, EVs can be modified to delay their clearance from circulation or improve their biodistribution. Royo et al. showed that neuraminidase treatment –a glycosidase that alters EVs surface glycosylation profile - induced an accumulation in the lungs compared with untreated EVs (Royo et al., 2019). Moreover, since macrophages can take up EVs and clear them from the circulation, EVs can be engineered through the incorporation of CD47—a transmembrane protein that enables macrophages evasion -, which initiates a “don’t eat me” signal (Imai et al., 2015; Kamerkar et al., 2017; Belhadj et al., 2020; Wei et al., 2021). Wei et al. reported that CD47-enriched EVs remained more time in circulation when compared with unmodified EVs (Wei et al., 2021). Similarly, the modification of EVs surfaces with polyethylene glycol (PEG) via PEGylation is another available strategy for improving EVs targeting (Clark et al., 2021).

Upon their arrival to the target tissue, EVs must be internalized by cells. There are several mechanisms by which EVs are up-taken by cells, depending on the specific type of EVs. These include clathrin-mediated endocytosis, caveolin-mediated endocytosis, macropinocytosis, phagocytosis, and direct fusion (Melling et al., 2019). Once incorporated into cells, EVs need to escape the endosomal compartment to prevent their degradation by lysosomes and cargo destruction. Some lysosomotropic molecules promote endosomal escape, such as chloroquine, amantadine, and ammonium chloride (Heath et al., 2019). Although the mechanisms underlying the endosomal escape of EVs are still not fully understood, Joshi et al. reported that EVs content release occurs from endosomes/lysosomes upon neutralizing endosomal pH and cholesterol accumulation to block EVs cargo exposure (Joshi et al., 2020). In addition, EVs can be engineered by expressing a pH-sensitive peptide, which allows EVs content release through the peptide fusion with the endosomal membrane upon endosomal acidification (Joshi et al., 2020). Nevertheless, additional efforts are needed to elucidate the mechanisms and the molecules involved in EVs processing after their incorporation by cells, in order to develop techniques that may improve cargo release.

### Upstream and Downstream Processing

In the production of EVs for therapeutic applications, regulatory agencies request a complete characterization of the active drug and its mode of action (MoA). They also require making batch-to-batch comparisons through specific biochemical, biophysical, and functional/potency assays. In this way, the early characterization of the manufacturing process is crucial since it enables the design of untimely standard operation procedures and the identification of potential risks and bottlenecks in advance, guaranteeing consistency and reproducibility (Rohde et al., 2019). EVs manufacturing process can be divided into two big stages: upstream processing –which encompasses all the operations required to produce the conditioned media (CM) for EVs isolation -, and downstream processing –which includes EVs purification and concentration, as well as the final product formulation and characterization (Staubach et al., 2021).

According to the International Society of Cell Therapy recommendations, it is critical to evaluate the parental MSCs to determine their phenotype, genetic stability, and potential biological contamination. While bacteria can be eliminated through filtration, viruses are more difficult to remove due to their similarity in size and charge with sEVs (Burnouf et al., 2019). In consequence, both *in vitro* and *in vivo* testing are required. Despite several methods are available in the literature for viral inactivation –including solvents, detergents, irradiation, and nanofiltration-, their utilization may alter EVs structure and content (Burnouf et al., 2019). Regarding MSCs tissue source, as many studies suggest, MSCs isolated from cancer patients may exert some pathological alterations, so the obtaining of healthy volunteers-derived allogeneic MSCs for EVs isolation is highly recommended (Hofer et al., 2010; Fernandez Vallone et al., 2013; Rühle et al., 2019). Open/semi-open systems have been the most utilized for cell culturing, although they may be associated with variability between batches, risk of contamination, and lack of real-time process control (Roura et al., 2017). In counterpart, closed systems employment is related to reduced risk of contamination, higher yields and cell-mass expansion, GMP compatibility, reduced costs of production, and the incorporation of in-process controls (Roura et al., 2017). Furthermore, it was demonstrated that 3D spheroidal culturing of MSCs promotes their exosome secretion (Kim M. et al., 2018; Yan and Wu, 2020). Multilayered cell culture flasks, hollow fiber bioreactors, stirred-tank bioreactors, and spheroidal aggregates of MSCs are among the most popular 3D-culture closed systems (Maumus et al., 2020). In this regard, it is necessary to establish a robust production system when using these technologies –which can be time-consuming - not only to control environmental parameters that may affect MSCs phenotype, but also to generate lot-consistent populations of EVs (Whitford and Guterstam, 2019). As previously mentioned, FBS represents some disadvantages when employed for clinical-grade MSCs culturing. Chemically defined media, human platelet lysate, and platelet-poor plasma xeno-free supplements have emerged as an alternative to FBS, with some contradictory associated reports. While platelet-poor plasma-cultured AT-MSCs showed impaired proliferative potential and an altered phenotype, MSCs from different sources cultured with xeno-free supplement derived from human plasma were reported to maintain their genetic stability, phenotype, and homogeneity (Blázquez-Prunera et al., 2017; Mushahary et al., 2018; Silva-Carvalho et al., 2020). However, these supplements hold some associated drawbacks. Human platelet lysate may contain platelet-derived contaminating EVs and pose the risk of pathogen contamination due to pooling –which is performed to limit batch-to-batch variability between donors - (Nyam-Erdene et al., 2021). In addition, the donor dependence for the obtainment of xeno-free supplements represents a limitation for industrial escalation of MSCs culturing. Moreover, since FBS removal may alter MSCs and MSC-derived EVs phenotype, EVs properties need to be corroborated after choosing a certain culturing media.

Concerning downstream processing, following CM harvesting, the first step includes multiple rounds of centrifugation or filtration to clarify the CM. Ultracentrifugation or density gradient centrifugation - used alone or in combination - are still considered the gold standards for sEVs purification and enrichment (Kain, 2005; Royo et al., 2020). Briefly, ultracentrifugation consists of a short centrifugation round at low speed to remove cells and cellular debris. Then, the supernatant is recovered and centrifuged 10,000–20,000xg for 20–30 min to pellet large ABs and MVs. sEVs remain in the supernatant and can be isolated by ultracentrifugation at high speed (100,000–120,000xg), with the time-lapse depending on the rotor size (Momen-Heravi, 2017). However, ultracentrifugation is associated with low yields, time-consuming centrifugation steps, mechanical damage to sEVs structure, and co-precipitation of contaminants - such as apolipoproteins A1/2 or B and albumin - (Busatto et al., 2018; Takov et al., 2019). Chromatographic techniques are commonly employed, including anion exchange chromatography, affinity chromatography, and size exclusion chromatography, which respective advantages and disadvantages are reviewed elsewhere (Staubach et al., 2021). Other novel approaches for sEVs purification encompass microchips, nanowires, and acoustic separation, as recently reviewed (Akbar et al., 2022). Similarly, new large-scale methods have been developed to improve EVs purification efficiency. For instance, tangential flow filtration is a novel technique that couples permeable membrane filtration and flow to obtain a concentrated sample of EVs from a colloidal matrix, and is more efficient, scalable, and involves less batch-to-batch variability than ultracentrifugation (Busatto et al., 2018). It requires a dead-end pre-filtration and a posterior step of membrane filtration (Gao et al., 2020). Zhang et al. also developed a purification strategy based on DNA aptamer-based magnetic isolation, mediated by CD63 binding (Zhang et al., 2019). However, as we previously mentioned, it is challenging to isolate highly pure populations of sEVs –this means the complete separation between exosomes and smaller MVs -. Using affinity chromatography with anti-CD63 antibodies may lead to the isolation of sEVs enriched in an exosome population, but the remaining antibodies or beads used for the purification process may interfere with further functional and potency assays by altering sEVs interaction with target cells (Tkach et al., 2018). When choosing a purification method, it is critical to compromise between EVs product purity and the method gentleness to maintain EVs functional properties. Another issue concerning EVs formulation is storage since it may alter EVs morphology, size, and particle concentration (Qin et al., 2020). After performing a comparative study, Wu et al. recommended storage at 4°C or −20°C for short-term preservation of sEVs and storage at −80°C for long-term preservation, in accordance with other studies (Herrmann et al., 2021; Wu et al., 2021). Since storage at −80°C implies high energy consumption and transportation issues, as well as repeatedly freeze and thaw cycles may alter EVs function, alternative storage conditions are being studied, such as lyophilization (Trenkenschuh et al., 2021).

After EVs purification, the final product needs to be characterized, as well as its safety, efficacy, and purity must be assessed through quality control procedures. EVs analysis is usually challenging due to the small size, biological complexity, and heterogeneity (Panagopoulou et al., 2020). As it happens with other biological and medical products, quality control procedures should evaluate identity, purity, potency, safety, and stability (Rohde et al., 2019). For instance, endotoxins from water, raw materials, equipment, and culture systems can contaminate EV formulations. Although affinity chromatography is the most used technique for endotoxin removal, novel and improved methods are being studied, including electronic biosensors and real-time monitoring systems (Schneier et al., 2020). EVs formulation purity is a standard normalization metric used to evaluate the product composition and to make comparisons between batches. It is essential for EVs dose determination and contaminant identification, and can be assessed through biophysical or biochemical methods. Regarding EVs characterization, nanoparticle tracking analysis - a method that tracks individual nanoparticles and derives their size and concentration in suspension– is used for EVs quantification and size determination, while electron-microscopy and cryo-electron microscopy are commonly used for EVs morphological characterization (Emelyanov et al., 2020; Comfort et al., 2021; Stam et al., 2021). Other methods used for EVs quantification include microfluidics, arrays, and polymerase chain reaction microfluidic systems (Panagopoulou et al., 2020). Multiplex bead-based assays are generally employed to evaluate and quantify EV surface markers, with EVs captured on antibody-coated beads coupled with flow cytometry read-outs (Wiklander et al., 2018). In this way, Kilic et al. recently developed a novel label-free multiplexed system to evaluate EVs surface protein profile through impedance spectroscopy (Kilic et al., 2022). Additionally, fingerprinting assays evaluate the presence or absence of a set of specific markers on EVs for quality control and determination of consistency between batches (Jeske et al., 2020). Finally, toxicity, safety, and efficacy require ultimate confirmation by the performance of qualified and well-standardized potency assays. These tests differ from functional assays since regulations define potency as “the specific ability or capacity of a product to effect a given result, as indicated by appropriate laboratory tests or by adequately controlled clinical data obtained through the administration of the product in the manner intended” (Nguyen et al., 2020). Although the US Food and Drug Administration recommends that potency assays should ideally represent the product MoA, this does not necessarily happen, because potency assays may not reveal information about EVs underlying mechanisms of action (Reiner et al., 2017; Gimona et al., 2021). Additionally, due to their biological complexity and, in consequence, their multifaceted MoA, the US Food and Drug Administration also recommends the utilization of an array matrix consisting of several potency assays for EVs evaluation (Gimona et al., 2021). Moreover, since EVs may exert multiple MoA given a specific pathological context, potency assays should be specifically related to the disease model for which they are intended (Willis et al., 2017). In conclusion, the development and standardization of improved clinical-grade quantification and characterization techniques, as well as potency assays for evaluating therapeutic EVs formulations, are critical requisites for EVs successful translation into the clinic as DDSs.

### Drug Loading

As previously demonstrated, MSC-derived EVs display high flexibility to modifications to improve their properties (Weng et al., 2021). This is a crucial feature for their usage as DDSs so that manufacturing processes frequently include endogenous or exogenous drug loading approaches. Regarding the endogenous approach, it involves the modification of the parental cell - through methods such as transfection/transduction or co-incubation of mainly small drugs with parental cells - followed by the purification of the modified MSC-derived EVs (Liao et al., 2019). The efficiency of the drug packaging into EVs depends mainly on its concentration inside cells (Susa et al., 2019). Transfection/transduction of parental MSCs leads to overexpression of mRNAs, proteins, or miRNAs. For instance, Li et al. cultured endometrial cancer cells with miR-302a-loaded EVs from modified UC-MSCs, showing a significant inhibition of the proliferation and migration of tumor cells through the blocking of AKT pathway (Li et al., 2019b). In the same way, Liu et al. demonstrated that BM-MSC-derived EVs containing let-7i miRNA –by modification of MSCs through a lentiviral vector transfection - inhibited lung cancer cells outgrowth both *in vitro* and *in vivo* (Liu J. et al., 2021). It is relevant to mention that viral testing - through high throughput techniques - has to be more exhaustive in those cases in which MSCs were genetically modified since the utilization of viral vectors may lead to insertional mutations and mutagenesis (Rohde et al., 2019). Some disadvantages are associated with endogenous drug loading, including low efficiency in RNA packaging into EVs and genetic instability in parental MSCs (Liao et al., 2019; Su et al., 2021). Thus, non-viral transfection methods began to be evaluated in MSCs, such as nanocarriers that interact with the plasma membrane, followed by cells uptake. These non-viral transfection methods are flexible and scalable, but showed reduced transfection efficiency as well as higher toxicities (Hamann et al., 2019). With reference to co-incubation methods, loading efficiency may be affected by drug properties, incubation periods, among others (Zhang X. et al., 2021), so co-incubation protocols should be optimized for each specific case.

On the other hand, through exogenous approaches, drugs are loaded into EVs after their purification by using different chemical and physical methods - which have to be analyzed in advance to evaluate potential alterations of EVs properties - (Herrmann et al., 2021). Among the most utilized techniques are co-incubation with drugs, electroporation, and sonication, while other methods such as cycles of freeze/thaw, EVs permeabilization with saponins, or extrusion, are less used (Zhao et al., 2020). Additionally, exogenous methods are classified into passive or active loading approaches. While the former includes co-incubation of EVs with hydrophobic drugs –whose loading efficiency depends on the drug gradient - the latter is frequently used for hydrophilic drugs that cannot spontaneously go across the EVs membrane (Herrmann et al., 2021). For example, Wei et al. reported the utilization of BM-MSC-derived EVs loaded with doxorubicin through passive co-incubation for osteosarcoma cells growth inhibition *in vitro* (Wei et al., 2019). Drug encapsulation efficiency –the mass of drug in EVs divided by the mass of drug added to the mix - is an important parameter that has to be determined when employing co-incubation methods since these techniques often result in low loading capacity (Villa et al., 2019; Li H. et al., 2022). In addition, it was reported that the lipid composition of the EVs may have an impact on the drug loading efficiency (Kooijmans et al., 2021). In counterpart, active loading methods can be classified into physical and chemical techniques. The former includes electroporation, sonication, and freeze/thaw cycles, while the latter involves using transfection reagents or saponins. Physical methods are characterized by a high loading efficiency compared with chemical methods, but may alter EVs membrane integrity and produce siRNA aggregation (Tang et al., 2019). Otherwise, chemical reagents may accomplish some toxicity levels (Tang et al., 2019).

### Surface Engineering

In order to improve MSC-derived EVs targeting properties and reduce their systemic toxicity, various EVs surface engineering approaches can be employed. On the one hand, genetic engineering of parental cells to display specific proteins on EV surfaces can only be utilized for protein and peptide engineering (Richardson and Ejima, 2019). In this way, Dooley et al. developed a platform for the fusion of proteins of interest to full-length or truncated forms of Prostaglandin F2 receptor negative regulator or Brain acid soluble protein 1, which are two scaffold proteins present in EVs and selectively sorted into them, allowing surface display and luminal loading of a wide range of molecules (Dooley et al., 2021). Yim N. et al. also developed a tool for exosomes engineering, named ‘exosomes for protein loading via optically reversible protein-protein interactions’ (EXPLORs). This system integrates a reversible protein-protein interaction module controlled by blue light –based on photoreceptor cryptochrome 2 protein - with the endogenous process of exosome biogenesis –through using a truncated version of Calcium and integrin-binding protein 1 conjugated to CD9 -. This method enables selective cargo of the therapeutic protein into EVs and its delivery to target cells after removing the illumination source (Yim et al., 2016). Similarly, the SMART exos system is based on expressing distinct types of monoclonal antibodies on sEVs surfaces that bind specific surface proteins in target cells (Shi et al., 2020). The genetic engineering approach can also be used to generate fusion proteins between the green fluorescent protein and sEVs-specific proteins –such as tetraspanins– to label sEVs for biodistribution analysis (Levy et al., 2021). A limitation of this method is that only a small population of sEVs become fluorescent, inducing variability in the signal intensity (Choi and Lee, 2016). Alternatively, specifically designed aptamers –single-stranded DNA/RNA oligonucleotides that can bind 3D structures and molecules with high affinity and specificity– can also be attached to EV surfaces through covalent linkage to improve their tumor tropism (Luo et al., 2019).

Regarding chemical approaches, EVs can be modified to anchor molecules or drugs on their surface through covalent linkage, PEG derivates, click chemistry techniques, and novel modification methods such as the utilization of ligases. Covalent linkage involves the employment of activated esters –i.e., hydroxysuccinimide esters - to link relatively simple molecules to the amino groups of EV surface proteins or anchor more complex biomolecules –such as antibodies– via biorthogonal reactions (Richter et al., 2021). In addition, nanobodies and other types of molecules such as lipid conjugates can be linked to EV superficial amino groups by using PEG derivates. These modified EVs showed an increased circulation time compared with unmodified ones (Kooijmans et al., 2016). Another popular approach is click chemistry –or azide-alkyne cycloaddition– which allows direct attachment of molecules to EV surfaces through covalent bonding. This method is simple with no impact on EVs structure, although it may alter EVs function by unspecific modification of proteins (Ramasubramanian et al., 2020). Pham et al. recently developed a covalent conjugation method of peptides or nanobodies to EV surfaces by employing protein ligases (Pham et al., 2021). They proved this method by anchoring an anti-epithelial growth factor receptor nanobody to EVs loaded with paclitaxel, which facilitated their tumor accumulation in a xenograft mouse model of epithelial growth factor receptor-positive lung cancer, and increased paclitaxel therapeutic efficacy (Pham et al., 2021). All these chemical methods require an additional purification step to prevent contamination of the final formulation with the reagents used for EVs surface modification (Richardson and Ejima, 2019).

**FIGURE 1:**
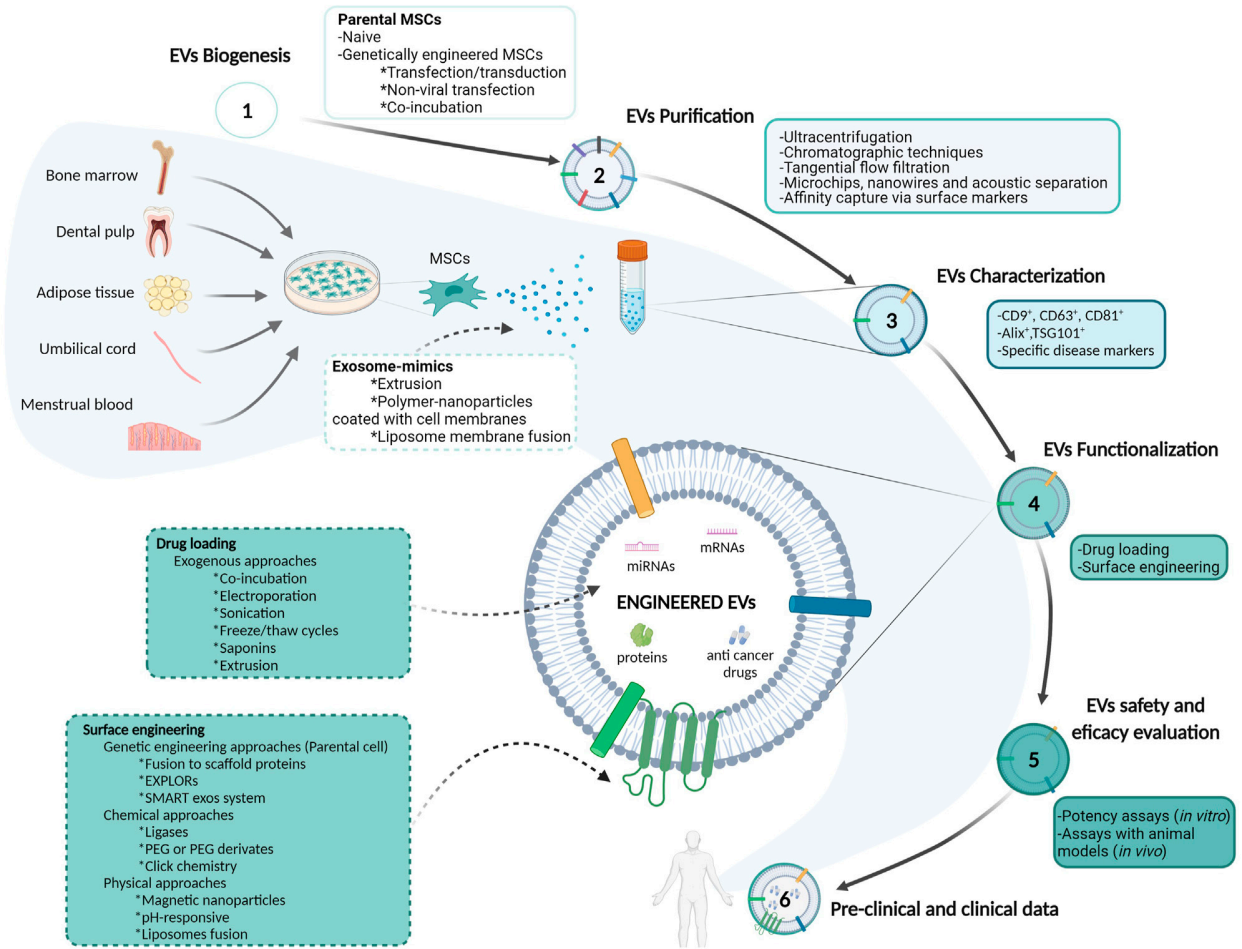
Schematic representation of mesenchymal stem/stromal cells-derived extracellular vesicles isolation, purification, characterization and evaluation approaches for their utilization as drug delivery systems in cancer therapy. Abbreviations: Alix: apoptosis-linked gene 2–interacting protein X; EVs: extracellular vesicles; EXPLORs: exosomes for protein loading via optically reversible protein–protein interactions; miRNA: microRNA; mRNA: messenger ribonucleic acid; MSCs: mesenchymal stem/stromal cells; PEG: polyethylene glycol; TSG101: tumor susceptibility gene 101 protein.

Furthermore, some physical approaches for EVs engineering have also received in-deep attention. For example, Mizuta et al. hybridized exosomes with magnetic nanoparticles to facilitate an efficient uptake by target cells after exposure to a magnetic field (Mizuta et al., 2019). Lee et al. also developed pH-responsive EVs containing hyaluronic acid grafted with 3-(diethylamino) propylamine and loaded with doxorubicin. These modified EVs responded to tumor pH (pH 6.5) and bounded to CD44 on HCT-116 human colorectal carcinoma cell line, inhibiting its growth *in vivo* (Lee et al., 2018). Finally, another emerging strategy is generating hybrid EVs by their fusion with liposomes. For instance, Piffoux et al. exploited the fusion of EVs –triggered by PEG - with functionalized liposomes, to enrich EVs with either lipophilic or hydrophilic drugs. These hybrid EVs showed no alteration of their structure and content and were more efficient in delivering chemotherapeutic compounds than their liposome precursor (Piffoux et al., 2018). All the information exposed in the previous sections is summarized in Figure 1.

### Exosome Mimics

Due to the limitation related to the amount of EVs that can be purified from *in vitro* culturing of parental cells, more significant numbers of nanovesicles can be produced through the implementation of certain emerging techniques, such as cell extrusion or the generation of polymer-nanoparticles coated with cell membranes, to obtain the so-called exosome-mimics (Li S.-p. et al., 2018) (Figure 1). Exosome-mimics are similar to natural sEVs in their properties, drug loading capacity, and interaction with target cells, but obtained at higher amounts and considered safer than natural sEVs (Vázquez-Ríos et al., 2019). Extrusion involves the sequential squeezing of cells through a set of extrusion filters to obtain nanosized or microsized vesicles that generally share membranes with their parental cells (Wang et al., 2021). In counterpart, polymer-nanoparticles coated with cell membranes can be obtained by mixing purified exosomes or isolated cell membranes with artificial polymer-nanoparticles (Tian et al., 2020). Additionally, the use of cytochalasin B was shown to simplify the large-scale production of exosome-mimics from MSCs, as this compound causes actin filament dissociation and cell disintegration after shaking the treated cells, with the formation of multiple vesicles that are built from cell plasma membrane (Chulpanova et al., 2021). Exosome-mimics showed higher half-life in the blood, prolonged circulation retention than PEG-coated nanoparticles, and reduced clearance by macrophages (Li S.-p. et al., 2018). Additionally, “artificial exosomes” can be generated from conventional liposomes by adding specific molecules. For instance, Haraszti et al. incorporated one lipid (dilysocardiolipin) and three proteins (Ras-interacting protein Rab7, Desmoplakin, and Alpha-2-HS-glycoprotein) —which they found to be enriched in serum-deprived MSCs - into neutral liposomes to produce vesicles that mimic the tropism and cargo delivering of natural sEVs (Haraszti et al., 2019). In this way, the extensive knowledge obtained from the EVs field can be applied to artificial nanocarriers engineering to improve their biological and therapeutic properties.

## Pre-Clinical and Clinical Data in Cancer

The first known references MSC-derived EVs for cancer cell-free therapy were published in 2013 (Katakowski et al., 2013; Ohno et al., 2013). Since then, many pre-clinical studies have been published describing the anti-tumor effects of engineered MSC-derived EVs. Here, we review some pre-clinical studies published between 2018 and 2022 (Table 1). They widely differ in the source of MSCs, the tumor models, and the approaches employed for cargo loading or EV surface engineering. For instance, Wang et al. developed genetically engineered murine BM-MSCs to overexpress the miR-185, which is known to attenuate inflammation associated with oral leukoplakia (Wang et al., 2019). In this way, similar approaches for EVs modification could be used to prevent the malignant transformation of potentially malignant oral disorders. In addition, many works reported the study of engineered MSC-derived EVs to target the known as the *hallmarks of cancer*. As Hanahan and Weinberg described in their renowned article in 2011, tumor cells exert some distinctive and complementary capacities that allow them to grow, invade and disseminate to distant organs. These characteristics include sustained proliferative capacity, apoptosis evasion, genomic instability, angiogenesis, replicative immortality, inflammation, metabolism deregulation and immune system evasion (Hanahan and Weinberg, 2011). Dong et al. treated human UC-MSCs with the siRNA anti-ELFN1-AS1 –a long non-coding RNA highly expressed in colon adenocarcinoma cells - and found that the EVs purified from siRNA-ELFN1-AS1-treated UC-MSCs could inhibit colon adenocarcinoma cells proliferation and migration *in vitro*. With reference to immune evasion, Zhou et al. demonstrated that miR-424-5p delivery through AT-MSC-derived EVs partly exerted pro-inflammatory effects and enhanced anti-tumor cytotoxicity in human triple-negative breast cancer cells both *in vitro* and *in vivo*, through the downregulation of the PD-L1 pathway (Zhou et al., 2021). Chulpanova et al. genetically modified human AT-MSCs to overexpress human IL-2. The engineered MSC-derived EVs were able to activate human CD8^+^ T cells, which in turn induced apoptosis in triple-negative breast cancer cells (Chulpanova et al., 2021). Furthermore, AT-MSC-derived EVs overexpressing miR-15a, which targets the demethylase KDM4 that is deregulated in colorectal cancer cells, were shown to diminish the immune evasion of tumor cells via the KDM4B/HOXC4/PD-L1 axis, both *in vitro* and *in vivo* (Liu L. et al., 2021). These results demonstrate that MSC-derived EVs may not only target the intrinsic tumor cells capacities, but also their ability to interact with the tumor microenvironment. Another study showed that the *in vitro* and *in vivo* delivery of miR-193a to colon cancer cells through BM-MSC-derived EVs inhibited their proliferation, migration and invasion through the Focal Adhesion Kinase targeting (Ying et al., 2020). This evidence supports the potential use of engineered MSC-derived EVs as a strategy for inhibiting the initial steps of the metastatic cascade. Finally, as we previously summarized in a previous review, there are some emerging pre-clinical strategies for targeting the establishment of the pre-metastatic niche (PMN) (Sanmartin et al., 2021). Although none of these mentioned therapeutic approaches consist of the utilization of MSC-derived EVs, these nanovesicles could be tested for the delivery of drugs that aim to target the PMN. For instance, some works described the use of antibodies to target soluble factors –such as the Dickkopf-related protein 1 and the C-C motif chemokine ligand 2– involved in bone/bone marrow PMN formation (Heath et al., 2009; Bonapace et al., 2014). Engineered MSC-derived EVs –particularly EVs isolated from BM-MSCs that exert tropism for bone/bone marrow PMN - carrying those antibodies in their surfaces could be developed as an improved strategy to alter the formation of the PMN and increase drug access to these niches.

**TABLE 1:** Pre-clinical data from the 2018-2021 period, regarding extracellular vesicles evaluation as drug delivery systems for cancer therapy. Abbreviations: AT-MSCs: adipose-tissue mesenchymal stem cells; BM-MSCs: bone-marrow MSCs; DMBA: 7,12-dimethylbenzathracene; DOX: doxorubicin; EVs: extracellular vesicles; GRP78: glucose-regulated protein 78; HCC: hepatocellular carcinoma; LNA: locked nucleic acid; LPS: lipopolysaccharide; MUC1: mucin 1 cell surface-associated; NSCLC: non-small cell lung cancer; OPMD: oculopharyngeal muscular dystrophy; PCNA: proliferating cell nuclear antigen; PDAC: pancreatic ductal adenocarcinoma; TRAIL: tumor necrosis factor-related apoptosis-inducing ligand.

Type of Parental MSCs	Modification Method	Tumor/Malignant Disorder Model	Effects	Reported by
Murine AT-MSCs	Pre-conditioning with LPS, and loading of anti-oncogenic miRNA-16-5p through membrane fusion with liposomes	E0771 and 4T breast cancer cell lines, both *in vitro,* and *in vivo* through mouse subcutaneous models	Decreased tumor cell proliferation and migration, and enhanced tumor cell apoptosis *in vitro*	Li et al. (2020)
Murine BM-MSCs	Genetically engineered MSCs to overexpress the anti-oncogenic miR-185 in EVs	Oral leukoplakia (buccal lesions in a DMBA-induced OPMD mouse model *in vivo*)	Attenuated inflammation severity, significantly decreased incidence and the number of dysplasia in the OPMD tissue *in vivo*, through inhibition of AKT and PCNA pathways	Wang et al. (2019)
Human MSCs cell line (Lonza)	Lentivirus-transfected MSCs to overexpress the tumor suppressor miRNA-584	Human glioblastoma cell line (U87)*,* and mouse xenografts	Reduced tumor cells proliferation, migration and invasion *in vitro*, and reduced tumor progression *in vivo*	Kim R. et al. (2018)
Human BM-MSCs	EVs loaded with paclitaxel	Human breast cancer cell line (MDA-MB-231) and subcutaneous mouse xenografts	Significantly decreased tumor cells viability *in vitro*, and inhibition of tumor growth *in vivo*, compared to naïve EVs	Kalimuthu et al. (2018)
Human UC-MSCs	EVs from pre-irradiated MSCs	Human malignant melanoma cell lines (A375 and G-361) and human breast cancer cell line (MCF-7), and their respective mouse xenografts	Decreased tumor growth *in vivo*, and significantly decreased number of metastatic foci *in vivo*	de Araujo Farias et al. (2018)
Murine BM-MSCs	Genetically engineered MSCs through a non-viral vector, to overexpress the anti-tumoral factor TRAIL	Subcutaneous mouse models of a mouse melanoma cell line (B-16-F0)	Reduced tumor size *in vivo*	Shamili et al. (2018)
Murine BM-MSCs	MSC-derived EVs loaded with DOX through electroporation. Surface engineering of EVs with carboxylic acid-end MUC1 aptamer	MUC1-positive murine colon carcinoma cell line (C26) and human breast cancer cell line (MCF-7)*,* as well as C26 mouse xenografts	Higher cytotoxicity *in vitro*, and suppression of tumor growth *in vivo*	Bagheri et al. (2020)
Human AT-MSCs	Lentivirus-transduced MSCs to overexpress miR-199a	Human HCC cell lines (Huh7, SMMC-7721, and PLC/PRF/5), and a PLC/PRF/5 orthotopic mouse model with DOX treatment	Increased HCC cells chemo-sensitivity to DOX (by inhibiting mTOR pathway) *in vitro* and *in vivo*, compared to the free drug	Lou et al. (2020)
Human BM-MSCs	Transfection of MSCs with oligonucleotides of miR-1231 mimics	Human PDAC cell lines (BxPC-3 and MIA PaCa-2) and BxPC-3 subcutaneous mouse xenografts	Inhibition of PDAC cells proliferation, migration and invasion *in vitro*. Suppression of tumor growth *in vivo*	Shang, et al. (2019)
Human MSCs cell line (Lonza)	Lentivirus-transduced MSCs to overexpress tumor suppressor miR-124a	A panel of human glioma stem cell lines (GSC267, GSC20, GSC6-27, GSC8-11, and GSC2-14), and intracranial mouse xenografts	Significantly reduced viability and clonogenicity *in vitro*, and increased overall survival of animal models	Lang et al. (2018)
Human UC-MSCs	Lipotransfection of MSCs with a miR-375 mimic	Human esophageal squamous carcinoma cell lines (KYSE70, ECA109, and EC9706, and subcutaneous KYSE70 and EC9706 mouse xenografts	Inhibition of cell proliferation, invasion, migration, and tumorsphere formation *in vitro*. Promotion of apoptosis *in vitro*. Inhibition of tumor growth *in vivo*	He et al. (2020)
Human AT-MSCs, BM-MSCs and UCB-MSCs	MSCs engineered to express the yeast cytosine deaminase::uracil phosphoribosyl transferase suicide fusion gene, through MSCs transduction with a recombinant retrovirus	Human glioblastoma cells obtained from primary tumors	Tumor cell growth inhibition *in vitro*	Altanerova et al. (2019)
Murine BM-MSCs	MSC-derived EVs loaded with LNA modified antimiR-142-3p molecules via electroporation	Human breast cancer cell line (MCF-7) mammospheres	Reduced clonogenicity and tumorigenicity *in vitro.* Induction of apoptosis *in vitro*	Naseri et al. (2020)
BM-MSCs cell line (ScienCell)	MSC-derived EVs loaded with paclitaxel (through sonication) and gemcitabine (through reversible electroporation)	Human PDAC cell line (MiaPaca-2 cells, tumor spheroids), and a MiaPaca-2 orthotopic mouse model	Increased homing and penetrating abilities *in vivo*, compared to the free drugs. Anti-tumor efficacy *in vivo* and *in vitro*	Zhou et al. (2020)
Human BM-MSCs	MSCs transfected with siRNA against GRP78	Human HCC cell lines (HepG2 and PLC), and HepG2 and PLC orthotopic subcutaneous and metastasis mouse models	Inhibition of Sorafenib-resistant HCCs growth and invasion *in vitro*. Inhibition of growth and metastasis *in vivo*	Li H. et al. (2018)
Human BM-MSCs	MSCs chemically transfected with a miR-199a mimic	Human glioma cell lines (U251, LN229, T98G, LN-18, SF-539 and A172) and U251 subcutaneous mouse xenografts	Inhibition of glioma cells proliferation, invasion and migration *in vitro*. Tumor growth inhibition *in vivo*	Yu et al. (2019)
Human BM-MSCs	MSCs chemically transfected with miR-144 mimic	Human NSCLC cell lines (A549, NCI-H1975, NCI-H1299), and NSCLC cell lines mouse xenografts	Inhibition of NSCLC cell proliferation and colony formation *in vitro*. Inhibition of tumor growth *in vivo*	Liang et al. (2020)
Human BM-MSCs	MSCs chemically transfected with a plasmid encoding miR-15a mimic	Human HCC cell lines (Hep3B and Huh7), and HCC mouse xenografts	Restriction of HCC cells proliferative, migrating, and invasive potentials *in vitro*. Promotion of HCC cells apoptosis *in vitro*. Reduced tumorigenicity and metastasis *in vivo*	Ma et al. (2021)
Human BM-MSCs	MSCs transfected with a lentivirus encoding miR-29a-3p mimics	Human glioma cell lines (U87MG and A172), and U87 mouse xenografts	Attenuated glioma cells migration and vasculogenic mimicry formation *in vitro*. Inhibition of tumor growth *in vivo*	Zhang Z. et al. (2021)

Other engineered MSC-derived EVs include the combination of AT-MSCs pre-conditioning with LPS to downregulate the expression of CD90, with the loading of anti-oncogenic miRNA-16-5p into CD90^low^-AT-MSC-derived EVs to enhance anti-tumoral effects (Li et al., 2020). Similarly, physical pre-conditioning techniques such as UC-MSCs irradiation were used to obtain MSC-derived EVs for melanoma treatment *in vivo*, showing promising results (de Araujo Farias et al., 2018). Furthermore, the usage of MSC-derived EVs as DDSs has been exploited to improve the biodistribution of chemotherapeutic drugs and reduce their severe systemic side effects. For example, paclitaxel has been previously associated with cardiotoxicity, myelosuppression, and neurotoxicity, so Kalimuthu et al. utilized MSC-derived EVs loaded with paclitaxel for the delivery of this chemotherapeutic agent to breast cancer cells while minimizing systemic adverse effects (Kalimuthu et al., 2018). Similarly, Shamili et al. isolated EVs derived from murine BM-MSCs overexpressing TRAIL and showed that the administration of the encapsulated form of TRAIL was better at reducing tumor size *in vivo* than the free drug (Shamili et al., 2018).

Regarding the clinical trials involving MSC-derived EVs for cancer therapy, only one phase I trial is registered at www.clinicaltrials.gov (NCT03608631). This clinical study is currently evaluating the best dose and associated side effects of MSC-derived exosomes loaded with KrasG12D siRNA (iExosomes) during the treatment of participants with metastatic pancreatic cancer bearing the KrasG12D mutation. Although gemcitabine has been the standard of care for this particular type of cancer, there is a poor response to this drug, leading to reduced overall survival (Long et al., 2011). Additionally, pancreatic cancer is a highly therapy-resistant tumor, even to available immunotherapies, due to its intrinsic low tumor mutational burden, immunosuppressive microenvironment, and acellular fibrous stroma that impairs drug access to the tumor (Bear et al., 2020; Stopa et al., 2020). In this way, MSC-derived EVs could be an exciting option to overcome drug access issues in pancreatic cancer patients and other cancer types with similar characteristics.

However, as Gupta et al. reviewed after performing a systematic analysis, there are many inconsistencies regarding the doses of EVs used in pre-clinical studies (Gupta et al., 2021). Moreover, most of the analyzed articles lack of references to the rationale of the dose selection and treatment frequency. While EVs doses range from 0.001 to 100 mg of EV protein per kg. of body weight –and the same variability happens upon the use of particle number as EVs quantity determination -, the frequency of administration of EVs ranges from 1 to 6 times (Gupta et al., 2021). In this way, potency units as qualitative measures derived from potency assays could be used to homogenize the assessment of EVs doses, as Dal Collo et al. discussed after the development of an *in vitro* assay that measures T-reg induction by MSC-derived EVs (Dal Collo et al., 2020). Tieu et al. also reported that over two-thirds of the analyzed pre-clinical studies involved the administration of a single dose of MSC-derived EVs, and only a 28% of the studies reported the administration of multiple doses (Tieu et al., 2020). Furthermore, the EVs dose selection can depend on the particular disease, since it was reported that the EVs therapeutic dose required for neurological diseases is lower than the dose required for other systemic inflammatory diseases (Gupta et al., 2021). In the previously mentioned clinical study testing MSC-derived exosomes loaded with KrasG12D siRNA on pancreatic cancer patients (NCT03608631), the EVs dose used is not mentioned, while the treatment periodicity involves the administration of up to three infusions of EVs. For instance, when comparing with MSCs, clinical trials using MSCs to treat patients with Covid-19 pneumonia reported the infusion of one million of MSCs per kg. of body weight with up to three infusions of cells (NCT04444271, NCT04713878, NCT04429763). Additionally, Pacienza et al. showed that the use of HUCPVC-derived sEVs (released by 1 × 10^6^ cells) in a rat lung preservation model had higher anti-inflammatory effects when compared with the administration of HUCPVCs (1 × 10^6^ cells, via the pulmonary artery) (Pacienza et al., 2020). These results encourage the use of MSC-derived EVs as cell-free therapies in replacement of the administration of MSCs. Finally, additional efforts are needed to standardize EVs dose determination protocols.

## Discussion

Anti-cancer drug development is a challenging field because not only tumors can develop both intrinsic and extrinsic mechanisms of drug resistance, but also antineoplastic drugs may be associated with low bioavailability and off-target effects. Although MSC-based therapy has been investigated for many years for its application for cancer treatment due to their biological properties –mainly their low immunogenicity and tumor tropism - many groups reported contradictory results regarding the pro and anti-tumoral effects of MSCs. Although these discrepancies among studies have been attributed to the differences between tissue sources and donors and the lack of standardized protocols for MSCs isolation and *in vitro* culturing, researchers may tread carefully when using MSCs for cancer cell therapy. Consequently, the utilization of MSC-derived EVs –either naïve or modified to improve their delivery and targeting properties– as cell-free DDSs allowed to overcome some of the drawbacks associated with cell therapy since EVs are static and do not reproduce or represent a mutational risk when administered to patients. Moreover, because of their inherent biological complexity, EVs display better tropism and biocompatibility when compared with artificial nanocarriers. However, there are still some limitations associated with EVs as DDSs. To begin with, as any biological product, EV formulations must fulfill the requirements of regulatory agencies in terms of purity, safety, efficacy, and batch-to-batch homogeneity. The exhaustively controlled manufacturing processes and quality control protocols needed to accomplish GMP and regulatory standards also enormously increase the cost of EV production. Due to EVs biological complexity, it may be difficult to assess if the lipid bilayer, their content, or both are responsible for their therapeutic effect. Furthermore, the procedures employed for EVs engineering require additional purification steps to eliminate potential reagent contamination, and most of those procedures tend to increase even more the final product cost and may result in low yields as well. Additionally, it has to be kept in mind that, when using MSC-derived EVs, researchers need to keep investigating their not yet fully understood effects on tumor progression. Nevertheless, there has been an exponential development of new approaches to overcome those methodical and cost-associated limitations. For instance, the emergence of hybrid vesicles –by the fusion of sEVs and liposomes– and exosome-mimics brought together “the best of both worlds”, shedding some light over the new emergent paradigm of biological nanovesicles as DDSs. In the last 4 years, many research groups have published pre-clinical data regarding the potential applications of engineered sEVs for cancer therapy, with some promising results. Since these reports are heterogeneous in terms of the engineering methods used, source of the parental MSCs, and the tumor models employed, new efforts are required to design and standardize protocols that will undoubtedly facilitate EVs translation to the clinic for theragnostic applications.
